# Supplementary material for: Biochemical Characterization of the Seed Quality of a Collection of White Lupin Landraces from Southern Italy
Source: Plants (Basel). 2024 Mar 10;13(6):785. doi: 10.3390/plants13060785 (PMC10974116; doi:10.3390/plants13060785)
Supplement: Supplementary file 1 [file plants-13-00785-s001.zip › plants-2850088-supplementary.pdf]

Supplementary Material

Biochemical Characterization of the Seed Quality of a Collection of White Lupin Landraces from Southern Italy

Alfio Spina [1], Stefano De Benedetti [2], Giuditta Carlotta Heinzl [2], Giulia Ceravolo [2], Chiara Magni [2], Davide Emide [2], Giulia Castorina [3], Gabriella Consonni [3], Michele Canale [1] and Alessio Scarafoni [2,*]

[1] Council for Agricultural Research and Economics (CREA), Centro di Ricerca Cerealicoltura e Colture Industriali, Corso Savoia 190, 95024 Acireale, Italy
[2] Department of Food, Environmental and Nutritional Sciences, Università degli Studi di Milano, 20133 Milan, Italy
[3] Department of Agricultural and Environmental Sciences, Università degli Studi di Milano, Via G. Celoria 2, 20133 Milan, Italy
* Correspondence: alessio.scarafoni@unimi.it

Supplemetary Table S1. Weight of one hundred seeds and main biochemical characteristics of the investigated white lupin genotypes. Results are reported as mean ± standard deviation referred to the seed Dry Weight (DW). The significance level was set at 0.05 and it is reported with the lowercase letters in the brackets; the same letter means the difference is not statistically significant. FRAP: Ferric Reducing Ability of Plasma; TIA: Trypsin inhibitory activity; nd: not determined.

Name	Hundred seeds weight (g)	Protein (g/kg DW)	Lipid (g/kg DW)	FRAP (mmol Eq $FeSO_4$/g)	Polyphenols (mg Eq Quercetin/g)	Phytic acid (g/kg DW)	Phosphorous (g/kg DW)	TIA (%)
**Leonforte 1**	61.07±1.77 (d)	312±19 (cdefg)	101±3 (abc)	116.5±4.9 (efg)	7.5±0.3 (bcd)	2.1±0.1 (a)	0.58±0.04 (c)	7.0±0.7 (gh)
**Leonforte 2**	61.11±2.84 (d)	312±47 (cdefg)	108±4 (a)	156.6±7.6 (b)	8.1±0.6 (b)	2.3±0.3 (a)	0.66±0.06 (abc)	-1.1±3.1 (h)
**Leonforte 3**	45.72±2.13 (e)	274±1 (fgh)	95±2 (abcdef)	139.8±5.9 (bcde)	7.3±1.3 (bcde)	2.3±0.1 (a)	0.65±0.04 (abc)	5.1±0.4 (gh)
**Leonforte 4**	35.21±0.08 (d)	309±6 (defg)	95±4 (abcdef)	129.7±13.7 (cdef)	7.6±0.3 (bcd)	2.7±0.1 (a)	0.75±0.03 (abc)	-1.8±1.8 (h)
**Leonforte 5**	71.85±0.04 (c)	311±8 (cdefg)	99±4 (abcd)	117.2±4.0 (efg)	7.0±0.1 (bcdef)	2.8±0.3 (a)	0.79±0.06 (ab)	28.4±2.5 (b)
**Leonforte 6**	56.85±1.26 (d)	343±4 (cdef)	107±6 (ab)	109.0±2.8 (fg)	6.1±0.4 (def)	3.0±0.1 (a)	0.84±0.03 (a)	19.6±4.2 (cd)
**Acireale**	76.53±0.27 (c)	302±1 (efgh)	86±3 (bcdefgh)	235.7±11.7 (a)	7.3±0.3 (bcde)	2.5±0.2 (a)	0.71±0.08 (abc)	19±3.1 (cde)
**Canicattini**	22.57±0.07 (mn)	379±22 (bcde)	94±6 (abcdef)	145.4±7.9 (bcd)	10.4±0.6 (a)	2.4±0.1 (a)	0.69±0.04 (abc)	11.7±1.3 (defg)
**Modica**	31.38±1.24 (gl)	390±23 (abcd)	75±4 (fgh)	125.9±5.8 (def)	7.1±0.1 (bcdef)	2.2±0.1 (a)	0.63±0.03 (bc)	16.5±3.5 (def)
**Scicli**	33.25±1.44 (gi)	428±1 (ab)	51±2 (i)	148.5±4.9 (bcd)	7.5±0.3 (bcd)	2.7±0.3 (a)	0.75±0.07 (abc)	16.5±2.1 (def)
**Grammichele**	63.45±0.78 (d)	328±24 (cdefg)	97±4 (abcde)	117.6±3.7 (efg)	6.3±0.4 (def)	2.3±0.3 (a)	0.65±0.07 (abc)	10.8±2.0 (efg)
**Calabria 1**	32.10±0.42 (gl)	447±29 (ab)	89±4 (abcdefg)	124.8±2.8 (def)	7.9±0.6 (bc)	2.1±0.1 (a)	0.59±0.04 (bc)	10.5±0.8 (fg)
**Calabria 2**	47.58±1.05 (e)	223±2 (h)	66±6 (hi)	153.8±4.2 (bc)	6.7±0.4 (bcdef)	2.3±0.4 (a)	0.64±0.08 (abc)	12.7±1.8 (defg)
**Calabria 3**	40.86±1.68 (gl)	463±15 (a)	81±8 (cdefgh)	98.3±1.0 (g)	5.7±0.1 (f)	2.4±0.3 (a)	0.68±0.03 (abc)	0.1±1.1 (h)
**Calabria 4**	32.69±0.44 (gl)	392±13 (abc)	80±6 (defgh)	117.8±4.0 (efg)	7.3±0.6 (bcde)	2.2±0.1 (a)	0.63±0.03 (bc)	2±1.4 (h)
**Puglia**	25.10±0.85 (d)	258±21 (gh)	71±6 (ghi)	128.8±0.3 (cdef)	6.6±0.6 (bcdef)	2.5±0.1 (a)	0.70±0.07 (abc)	-0.5±2.1 (h)
**Lecce**	105.00±2.8 (a)	324±2 (cdefg)	107±7 (ab)	98.4±4.8 (g)	5.9±0.3 (ef)	2.5±0.3 (a)	0.71±0.01 (abc)	25.9±4.4 (bc)
**Basilicata**	34.30±0.42 (fh)	468±33 (a)	77±6 (efgh)	118.3±2.4 (efg)	6.4±0.1 (cdef)	2.4±0.1 (a)	0.75±0.03 (abc)	-2.4±3.7 (h)
**Molise**	87.05±3.23 (b)	283±28 (fgh)	75±6 (fgh)	138.7±8.9 (bcde)	6.1±0.1 (def)	2.3±0.3 (a)	0.64±0.03 (abc)	-1.5±3.5 (h)
**Ares**	35.58±1.26 (d)	394±5 (abc)	101±8 (abcd)	112.4±6.2 (fg)	2.8±0.2 (g)	2.6±0.2 (a)	0.70±0.07 (abc)	65.7±0.3 (a)

	Proteins	Lipids	TIA	FRAP	Polyphenols	Phytic Acid	P
**Proteins**	1.000						
**Lipids**	-0.169	1.000					
**TIA**	0.076	0.277	1.000				
**FRAP**	-0.255	-0.227	-0.081	1.000			
**Polyphenols**	-0.046	-0.065	-0.562	0.356	1.000		
**Phytic Acid**	-0.037	0.175	0.391	-0.026	-0.218	1.000	
**P**	0.074	0.132	0.349	-0.056	-0.248	0.966	1.000

Color range
1.000
0.750
0.500
0.250
0
-0.250
-0.500
-0.750
-1.000

Supplementary Figure S1. Correlation matrix: +1 = strong positive correlation (green); -1 = strong negative correlation (red). Table highlights a strong positive correlation between phosphorous and phytic acid content, and a positive correlation between polyphenol content and reducing power.
